# Unconventional Pathways of Secretion Contribute to Inflammation

**DOI:** 10.3390/ijms18010102

**Published:** 2017-01-05

**Authors:** Michael J. D. Daniels, David Brough

**Affiliations:** Division of Neuroscience and Experimental Psychology, Faculty of Biology, Medicine and Health, University of Manchester, AV Hill Building, Oxford Road, Manchester M13 9PT, UK; michael.daniels@postgrad.manchester.ac.uk

**Keywords:** unconventional secretion, nuclear localisation, inflammasome, IL-1β, IL-1α, IL-33, HMGB1

## Abstract

In the conventional pathway of protein secretion, leader sequence-containing proteins leave the cell following processing through the endoplasmic reticulum (ER) and Golgi body. However, leaderless proteins also enter the extracellular space through mechanisms collectively known as unconventional secretion. Unconventionally secreted proteins often have vital roles in cell and organism function such as inflammation. Amongst the best-studied inflammatory unconventionally secreted proteins are interleukin (IL)-1β, IL-1α, IL-33 and high-mobility group box 1 (HMGB1). In this review we discuss the current understanding of the unconventional secretion of these proteins and highlight future areas of research such as the role of nuclear localisation.

## 1. Introduction

### 1.1. Unconventional Protein Secretion

The classic dogma of protein secretion involves processing through the endoplasmic reticulum (ER) and Golgi body before secretion from the cell [1]. This process is dependent on the presence of an N-terminal “leader sequence” (also known as a “signal peptide”) which facilitates linking of the ribosome to the ER and translocation of the peptide [2]. This process of conventional secretion forms the most common way by which proteins leave the cell. However, a number of secreted proteins have been discovered which lack leader sequences and thus do not follow this pathway. Classical examples include fibroblast growth factor 2 (FGF2) [3] and the galectins [4]. These proteins follow an unconventional process of secretion through a variety of mechanisms and are often involved in essential processes such as tissue organisation, cell survival and immune regulation [5,6].

FGF2, also known as basic fibroblast growth factor, is a mitogenic factor that acts primarily by binding to high-affinity cell-surface receptors FGFR1-4 [7]. FGF2 lacks a leader sequence and thus is actively secreted by live cells through an unconventional pathway [8]. It is now understood that FGF2 is secreted by a mechanism dependent on interaction with the lipid membrane. Following binding to the phospholipid phosphatidylinositol 4,5-bisphosphate (PIP2), FGF2 is able to translocate directly through the cell membrane via formation of a pore dependent on Tec kinase activation [9]. FGF2 is then captured and ‘pulled out’ of the membrane pore by heparan sulphate chains to which it binds with strong affinity thus leading to its non-conventional secretion [10].

The above mechanism of capture by cell surface proteins is also reflected by the unconventional secretion of the galectins. The galectin family of proteins are β-galactose-recognising lectins involved in cell adhesion, promotion of cell-cell interaction, cell growth and apoptosis [11]. It has been known for over 25 years that galectin-1 (gal-1) can be secreted from the cell despite the lack of an N-terminal leader sequence [12]. However, it is only more recently that the mechanism behind gal-1 secretion is better understood. Gal-1 is secreted from cells by binding to β-galactosidase counter receptors on the cell membrane [13]. Evidence for this lies in the observation that mutants lacking the β-galactoside binding sites and the galectin counter receptors are deficient in gal-1 secretion. Some unconventionally secreted proteins such as IL-1β (discussed further below) utilise microvesicles or exosomes for their unconventional secretion [14]; however, this is not the case with FGF2 or gal-1 [15] which directly cross the plasma membrane.

### 1.2. Inflammation

Inflammation is our bodies’ response to infection or injury and is generally beneficial, neutralising the pathogen, and promoting repair and recovery. However, inflammation that occurs in the absence of a pathogen, during non-communicable diseases such as Alzheimer’s, diabetes or stroke is damaging and can make the outcome of the disease worse [16]. Inflammation in cases such as this is regarded as sterile and is now recognised as a therapeutic target. Inflammatory responses are regulated in part by cytokines; proteins that are actively secreted from cells which bind specific receptors to initiate inflammation. An inflammatory response can also be induced by factors called damage associated molecular patterns (DAMPs). DAMPs are endogenous molecules stored within cells that are released and invoke an inflammatory response following stress. Cytokines and DAMPs are key regulators of inflammation and can be secreted via unconventional pathways. Here we review four of these inflammatory mediators: IL-1β, IL-1α, IL-33 and HMGB1 and discuss current understanding of the mechanisms of their regulation and secretion.

## 2. IL-1β

Perhaps the best-studied of these inflammatory factors is the cytokine interleukin-1β (IL-1β). IL-1β is one of 11 members of the IL-1 family and acts as a crucial regulator of sterile inflammation in addition to host responses to infection. Unlike conventionally secreted cytokines such as IL-6, IL-1β does not have a leader sequence required for secretion through the ER/Golgi pathway and is secreted through unconventional means [17]. IL-1β is produced as an inactive 31 kDa pro form by myeloid cells (such as monocytes and macrophages) in response to activation of membrane bound pattern recognition receptors (PRRs) such as Toll-like receptors (TLRs) which detect pathogen associated molecular patterns (PAMPs, e.g., bacterial endotoxin), or DAMPs to initiate inflammation. This upregulation of pro-IL-1β expression is known as a priming step and leads to production of cytokine distributed across the cytosol [18].

**Figure 1:**
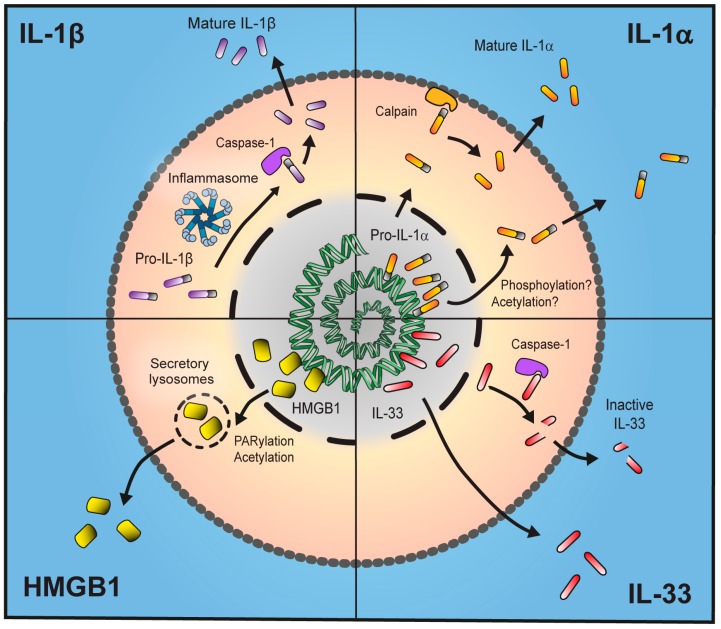
Unconventional secretion of inflammatory proteins. Interleukin (IL)-1β is produced as an inactive 31 kDa pro-form which, upon immune stimulus and activation of the inflammasome, is cleaved into a mature 17 kDa form and secreted from the cell. IL-1α is localised to the nucleus due to the presence of a nuclear localisation sequence (NLS). Following stimulus IL-1α is transported out of the nucleus, possibly by phosphorylation or acetylation, and either leaves the cell in its bioactive 31 kDa form or is processed to a more potent 17 kDa form by membrane-associated calpains. IL-33 also contains an NLS and thus is stored in the nucleus. It is fully active as a 33 kDa form and processing by caspase-1 leads to production of an inactive protein. High-mobility group box 1 (HMGB1) is stored in the nucleus but, following stimulus, is translocated into secretory lysosomes in the cytosol by Poly(ADP)-ribose polymerase (PARP)-1-dependent acetylation where it can be secreted either actively by live cells or passively due to necrosis.

Pro-IL-1β remains cell-associated until a second stimulus (a further PAMP or DAMP) triggers processing and secretion of 17 kDa mature IL-β which is dependent on the formation of a multi-molecular complex called an inflammasome (Figure 1). Inflammasomes are large (~1 μm) intracellular protein complexes that comprise a cytosolic PRR, adapter molecule and the enzyme caspase-1 which cleaves inactive pro-IL-1β to a mature form which is secreted from the cell [19]. The best characterised inflammasome is known as the NLRP3 inflammasome, so-called because the PRR molecule is nucleotide-binding oligomerisation domain (NOD)-like receptor, pyrin-containing 3. Upon activation by stimuli that induce potassium efflux from the cell [20], NLRP3 recruits the adapter molecule ASC (apoptosis-associated speck-like protein containing a carboxy-terminal CARD) to form a complex. The ASC molecule forms large prion-like filamentous structures known as specks [21] and recruits the 45 kDa zymogen pro-caspase-1 which is cleaved into an active IL-1β-processing form [22]. In addition to this classical activation of NLRP3 there also exists a “non-canonical” inflammasome activation pathway dependent on caspase-11 in mice and caspase-4 and 5 in humans [23].

IL-1β is secreted through an unconventional pathway that is not fully understood. There are multiple suggested modes of IL-1β release. Perhaps the best characterised mode of release is secretion tightly coupled to pyroptotic cell death. Pyroptosis is a form of inflammatory cell death characterised by the concomitant release of IL-1β and IL-18 [24] and can be induced by NLRP3-activating stimuli such as ATP binding to the extracellular ATP sensor P2X7 [25,26]. The activation of the NLRP3 inflammasome relies on association with the serine-threonine kinase NEK7 which forms a complex along with NLRP3, downstream of the crucial potassium efflux step [27,28]. Exactly how closely coupled cell death and IL-1β release are remains a major research question. Early research in macrophages suggested IL-1β was secreted before cell death as measured by release of lactate dehydrogenase [25,29]. More recent evidence, however, has clarified that membrane permeability (indicated by uptake of DNA dye propidium iodide) is required for IL-1β secretion in macrophages [30]. In human monocytic THP-1 cells, necrotic cell death is suggested to be required for IL-1β secretion by multiple stimuli [31]. However, primary human monocytes secrete IL-1β without loss of viability [32]. Death-independent inflammasome activation also appears to occur in the case of mouse dendritic cells [33] and neutrophils [34]. These data indicate that IL-1β can be secreted both in an active mechanism independent of cell death but also in a manner that absolutely requires loss of membrane integrity.

The process by which IL-1β is secreted by pyroptotic cells remained a mystery until last year when two groups used genetic screening methods to identify the N-terminal domain of gasdermin-D as a crucial regulator of pyroptosis [35,36]. It has since been discovered that the N-terminal cleavage product of gasdermin-D translocates to the plasma membrane and forms 10–14 nm pores in the membrane to induce pyroptosis [37,38,39]. In this way IL-1β is secreted through a mechanism not completely dissimilar to FGF2 discussed previously as the gasdermin molecules may allow for IL-1β to directly cross the plasma membrane whilst FGF2 forms a pore required for its own secretion. It is not fully understood why cells undergo pyroptotic death. One recent suggestion is that pyroptotic cells can retain bacteria within their corpses, structures called pore-induced traps (PITs) which are then cleared by secondary phagocytes [40]. This process could provide an evolutionary explanation for pyroptosis.

Human monocytes can secrete IL-1β in endolysosomal vesicles following treatment with ATP or hypotonic conditions [41]. Additionally, IL-1β secretion has also been observed in exosomes following P2X7 receptor stimulation on macrophages [42,43]. IL-1β release is commonly modelled using both “priming” and “activating” steps as described previously. However, inflammasome activation has also been observed with TLR4 stimulus alone in monocytes [44]. Stimulation of human monocytes with TLR4 ligand lipopolysaccharide (LPS) alone elicits ATP release from the cell which then acts back on membrane-P2X7 receptors in an autocrine mechanism to induce inflammasome activation and IL-1β release [45]. A similar form of “one-step” inflammasome activation and IL-1β release has also been observed in a caspase-5 dependent manner in human monocytes which appeared independent of cell death [32]. 

IL-1β secretion is also strongly associated with autophagy, a fundamental process through which organelles/proteins are “recycled” in a double-membrane vesicle called an autophagosome [46]. The precise role of autophagy in IL-β secretion is complex. Some studies suggest a tonic inhibition of IL-1β secretion by both degrading pro-IL-1β and by negatively regulating the NLRP3 inflammasome [47]. Conversely, additional research proposes that mature IL-1β is actively packaged into autophagosomal vesicles and secreted following stimulation of autophagy [48,49].

NLRP3-dependent secretion of IL-1β is closely linked to numerous non-communicable diseases including haemorrhagic disorders [50,51], Alzheimer’s disease [52,53], gout [54], diabetes [55] and atherosclerosis [56]. Aberrant inflammasome activity is also involved directly in a family of genetic diseases known as cryopyrin-associated periodic fever syndromes (CAPS) in which gain-of-function mutations lead to spontaneous NLRP3 activation and IL-1β secretion [57].

Due to the crucial involvement of NLRP3 in sterile disease, but the relative lack of importance in host-defence [58] the inflammasome has become a highly attractive target for therapeutic inhibition. There have been multiple compounds shown to inhibit NLRP3-induced IL-1β secretion (reviewed by [59]). The most NLRP3 inhibitor is MCC950, a diarylsulfonylurea-containing compound initially discovered by Pfizer [60] and since shown to be a potent and specific inhibitor of NLRP3 [61]. Currently available drugs can also be repurposed to inhibit NLRP3. Nucleoside reverse transcriptase inhibitors used to treat human immunodeficiency virus (HIV) inhibit P2X7-induced NLRP3 activation in a model of age-related macular degeneration [62], whilst Daniels et al. [63] showed that the fenamate non-steroidal anti-inflammatory drug (NSAID) mefenamic acid is a specific NLRP3 inhibitor and is effective in rodent models of Alzheimer’s disease.

## 3. IL-1α

IL-1α is also a member of the IL-1 family and is secreted through unconventional pathways. Although a major mediator of inflammation, IL-1α remains relatively poorly researched when compared to IL-1β. IL-1α was discovered along with IL-1β as a pyrogen [64] and is found at the same locus on chromosome 2 in the region q13 to q21 in humans suggesting it occurred as a result of a duplication event [65]. Like IL-1β, IL-1α is involved in a number of disease states including stroke [66], haemorrhage [67], cancer [68], and atherosclerosis [69]. IL-1α also plays a key role in promoting cellular senescence, a state of permanent cell-cycle arrest undergone by cells as a result of ageing or conditions of cell stress [70,71]. IL-1α regulates senescence by promoting the senescence-associated secretory phenotype (SASP), a response characterised by secretion of IL-6 and IL-8 [72].

Unlike IL-1β, whose expression is generally inducible, IL-1α is reported to be constitutively expressed at low levels in various cell types such as fibroblasts [73] and keratinocytes [74]. However, the bulk of the literature has been performed in myeloid cells and suggests that IL-1α expression is inducible and only observed following stimulation [66,75,76,77]. The constitutive expression of IL-1α is believed to be regulated by the transcription factor Sp1 which binds between −52 and −45 bp at the 5′ end of the *IL1A* gene [65,78]. Inducible IL-1α expression on the other hand relies primarily on the AP-1 and NF-κB transcription factors [79,80] thus explaining the overwhelming evidence for induction of IL-1α expression following immune stimulus. An additional mechanism regulating IL-1α expression occurs in the form of a long noncoding (lnc)RNA located on the anti-sense strand of the gene [81]. This lncRNA—itself induced by immune stimulus—has been shown to be crucial in promoting transcription and expression of IL-1α in murine macrophages.

In addition to a differing expression pattern in terms of cell types and induction, IL-1α can also be distinguished from IL-1β by its sub-cellular distribution. Unlike IL-1β, pro-IL-1α contains a nuclear localisation sequence (NLS) in the N-terminal pro-piece [82] and thus is found within the nucleus of the cell (Figure 1). NLSs are the best understood mechanism by which cells transport cargo in and out of the nucleus. Transport through the nuclear envelope is regulated by the karyopherin-β (kapβ) family of transport receptors which target short motifs of basic amino acids (the NLS) for nuclear import [83]. The NLS on pro-IL-1α is a highly conserved classical monopartite sequence consisting KVLKKRRL (human) and KILKKRRL (mouse) at residues 79–86 [82]. Although the presence of the highly conserved NLS on pro-IL-1α has been known for over 30 years, the precise role the motif plays in IL-1α secretion or signalling remains poorly understood. It has been suggested that the N-terminal pro-piece of IL-1α activates transcription of pro-inflammatory genes [84,85], thus maintaining an overall pro-inflammatory function. However, there is also evidence suggesting that the NLS of pro-IL-1α may be anti-inflammatory in nature. It was observed that pro-IL-1α is actively trafficked to the nucleus to dampen inflammation in apoptotic [86] or necrotic cells [18,87].

The mechanisms that regulate nuclear trafficking of pro-IL-1α are uncharacterised. Early research suggested that changing phosphorylation states on crucial lysine residues of the NLS regulates intracellular transport [88,89]. More recent evidence has also proposed that acetylation regulated by histone deacetylase (HDAC) enzymes positively regulates nuclear redistribution [90] implying that post-translational modifications may play a crucial role in pro-IL-1α nuclear shuttling. Further research is required in order to fully elucidate the importance of the NLS in IL-1α signalling/release.

IL-1α functions primarily as a pro-inflammatory cytokine by binding IL-1R1 and activating a MyD88-dependent pathway resulting in NF-κB, c-Jun N-terminal kinase (JNK) and p38 signalling cascades similar to IL-1β [91]. Again similar to IL-1β, IL-1α is produced as a 31 kDa pro- form which contains no leader sequence to target it for conventional protein secretion. Unlike IL-1β however, there is evidence that the pro-form of IL-1α is biologically active [92,93], although the physiological significance of this is yet to be fully understood. Some research has suggested that cleavage of pro-IL-1α into a 17 kDa form renders the cytokine far more active at IL-1R1 [94,95]. However, there is also evidence to suggest that the pro and cleaved forms have similar bioactivity [96]. Cleavage of IL-1α appears to be primarily regulated by calcium-dependent proteases known as calpains [97,98] (Figure 1). This is suggested by evidence showing that both Ca^2+^-free conditions and calpain inhibitors prevent IL-1α processing and release from macrophages [96,99]. However, the specific calpain required for pro-IL-1α cleavage is not known. The calpain family is made up of 14 distinct members and the best studied are calpain-1 and calpain-2 [100]. These currently stand as the most likely candidates for IL-1α processing. Calpains classically perform enzymatic cleavage at the inner leaflet of the plasma membrane tethered to phospholipids [101,102]. IL-1α has been reported to also bind to phospholipids on the inner-membrane of the cell in a Ca^2+^-dependent manner [88] suggesting that cleavage may take place following translocation of IL-1α to the lipid membrane. More recently, calpain activation has been implicated not only in IL-1α processing but in a number of members of the P2X7-induced secretome including IL-1β [103].

In addition to cleavage of pro-IL-1α by calpains, the cytotoxic lymphocyte-derived protease granzyme B is also known to cause IL-1α processing [95]. Functional cleavage by elastase or chymase was also reported in the above study.

The exact mechanism by which IL-1α leaves the cell is poorly understood although it does appear that IL-1α release is associated with cell death. Cohen et al. [86] observed IL-1α secretion from necrotic, but not apoptotic cells. Additionally, the process of necroptosis, a caspase-independent, RIPK-dependent form of programmed necrosis leads to IL-1α secretion [99]. IL-1α is also secreted in a caspase-11-dependent manner in cases of non-canonical inflammasome activation [23]. IL-1α release following non-canonical inflammasome activation, however, appears independent of NLRP3 or potassium ion efflux (unlike IL-1β secretion) [104]. The link between NLRP3 and IL-1α secretion was also explored by Gross et al. [96] who discovered that, whilst all inflammasome-activating stimuli induced secretion of IL-1α, only ATP, nigericin and *candida albicans*-induced secretion was NLRP3-dependent, whilst particulate stimuli such as monosodium-urate (MSU) crystals induced IL-1α secretion independently of NLRP3. In addition to cell-death dependent IL-1α secretion, IL-1α can also be expressed on the cell membrane independently of cell death where it plays a crucial role in driving the senescence-associated secretory phenotype (SASP) [71]. However, the mechanism by which this occurs is not understood. There is also some evidence that IL-1α secretion precedes cell death [96] and that secretion can occur at a basal (but very low) level in macrophages [105] or aged fibroblasts [106].

The biological relevance of IL-1α nuclear localisation is poorly defined, and nuclear localisation has been reported to both promote and abrogate inflammation. IL-1α can also be secreted/released from the cell as a cytokine/DAMP. The mechanisms by which IL-1α is secreted appear complex, varied and poorly understood. It would appear that by being constitutively expressed in non-myeloid cells and active in its pro-form, IL-1α can act as a DAMP when released under necrotic conditions. However, following induction of expression in immune cells, active processing by calpains and binding to IL-1R1, IL-1α also functions as a classical cytokine.

## 4. IL-33

In addition to IL-1α and IL-1β, other members of the IL-1 family have been reported as DAMPs including IL-33 [107]. IL-33 was first discovered as a nuclear protein and initially named NF-HEV (nuclear factor from high endothelial venules) [108]. IL-33 has been implicated in numerous disease states including respiratory disorders such as asthma [109,110] and COPD [111] in addition to arthritis [112,113]. Like IL-1α, IL-33 is constitutively expressed in multiple cell types and is not required to be induced by a priming stimulus [114,115].

The first identification of IL-33 was of a nuclear protein thought to be preferentially expressed in high endothelial venules (HEVs), structures involved in lymphocyte recruitment [108]. Baekkvold et al. [108] also identified the classical bipartite nuclear localisation sequence on IL-33. In a following study, the same group rediscovered IL-33 as the IL-1 family ligand for the orphan receptor ST2 and observed that the protein associates with heterochromatin and mitotic chromatin in both human and mouse cells [116]. The precise role played by intranuclear IL-33 is not fully understood. However, it appears that the nuclear localisation is anti-inflammatory. Evidence for this lies in early discoveries that IL-33 appears to aid chromatin compaction and repress gene expression [116,117]. Additionally, further studies suggested that the N-terminus of IL-33 may dampen NF-κB signalling by associating with the NF-κB p65 subunit and preventing binding of p65 to target DNA sequences [118]. Perhaps most strikingly, genetic deletion of the classical bipartite nuclear localisation sequence in mice leads to a lethal IL-33-mediated inflammatory response, suggesting nuclear localisation is vital for regulating aberrant inflammatory activity [119].

IL-33 elicits extracellular effects by binding to the ST2 receptor [120]. The C-terminus of the protein, along with the IL-1 receptor accessory protein (IL-1RAcP) forms a complex with the ST2 receptor in order to initiate a type 2 inflammatory response [121]. Type 2 responses are classically associated with injury resolution and helminth infection and characterised by secretion of Th2 cytokines such as IL-4, IL-5, IL-9, IL-10, and IL-13 [122]. Following formation of the receptor complex, MyD88, IRAK, IRAK4, and TRAF6 are all recruited to ST2 leading to a downstream activation of NF-κB, JNK and mitogen-activated protein kinase (MAPK) signalling [120].

Upon the initial discovery of IL-33 it was suggested that, like IL-1β, caspase-1 is required for processing of a 31 kDa pro-IL-33 to a 20–22 kDa mature, biologically active form [120]. It has since been clarified, however, that the 20–22 kDa caspase-1 cleavage product in fact corresponds to the N-terminal domain (which does not bind or activate the ST2 receptor) and that the full-length 31 kDa form of IL-33 is fully biologically active [123] (Figure 1).

Like IL-1α, IL-33 is secreted from cells undergoing necrotic death. This includes death as a result of physical damage in human endothelial cells [123], detergent-based lysis in mouse macrophages [124] or by parasite-induced necrosis [125]. Cell damage/death is not always required for IL-33 secretion. Mouse astrocytes treated with recombinant TNFα actively secrete IL-33 without loss of cellular viability [126] whilst normal human bronchial airway epithelial cells treated with extract of the common airway allergen *Alternaria alternata* also secrete IL-33 without compromising cell integrity [127]. *Alternaria*-induced IL-33 secretion independent of cell death occurs as a result of a P2Y receptor-dependent calcium influx which is caused by autocrine-mediated ATP stimulation, similar to autocrine IL-1β release mentioned previously [45]. In this way, cells can secrete IL-33 in both active mechanisms dependent on certain stimuli or in passive mechanisms through cell lysis.

The precise mechanisms underpinning IL-33 secretion remain largely undefined. The fact that IL-33 is constitutively expressed and that it is active as a pro form, much like IL-1α, suggests that it primarily functions as a classical DAMP. Further research is required to fully elucidate exactly which conditions result in active secretion of IL-33 (a cytokine role) versus its release as a DAMP during cell death, and why this occurs.

## 5. HMGB1

Unconventionally secreted inflammatory modulators do not just come from the IL-1 family. In fact, the ability to invoke an inflammatory reaction in the absence of classical secretion methods is highly conserved and found in multiple species across the animal, plant, protozoan and fungus kingdoms [128]. The best studied example of this is the phylogenetically ancient protein high-mobility group box 1 (HMGB1).

HMGB1 was initially discovered and characterised as a nuclear protein after it was found to co-precipitate with chromosomal DNA. Nuclear localisation of HMGB1 occurs due to the presence of a complex set of localisation signals located both at amino acids 27–43 and 178–184 [129]. Within the nucleus, HMGB1 acts to regulate gene expression. Here it binds loosely to DNA (distinguishing it from tightly bound histones) and facilitates DNA bending. This process allows binding of regulatory complexes such as V(D)J recombinases (responsible for generation of the diverse repertoire of immunoglobulins and T-cell receptors) [130] or nuclear hormone receptors [131]. Indeed, the importance of the nuclear role of HMBG1 is perhaps best illustrated by the fact that gene knockout is lethal in mice as a result of disruption of gene transcription, induced by the glucocorticoid receptor [132].

Since it was first described in 1976 [133] HMGB1 was known only for its nuclear role described above. However, more than 20 years following this, a crucial additional role for HMGB1 outside of the cell was discovered [134]. HMGB1 is secreted by macrophages in the later stages of sepsis through an unconventional pathway (Figure 1). Moreover, administration of neutralising antibodies to HMGB1 is protective against endotoxin induced lethality. Following this discovery, the cytokine role of HMGB1 has been further confirmed in numerous studies linking to diseases such as sepsis [135], lung disease [136], arthritis [137], stroke [138,139] and haemorrhagic shock [140].

Following its secretion, HMGB1 promotes an inflammatory response. Although no specific HMGB1 receptor has been identified (unlike IL-1β), it would appear that downstream effector functions occur following binding to polygamous receptors such as the receptor for advanced glycation endproducts (RAGE), TLR2 or TLR4. The first receptor reported to confer downstream effects of HMGB1 was RAGE [141,142]. Upon binding RAGE, HMBG1 initiates activation of NF-κB leading to production of classical proinflammatory cytokines [143]. HMGB1 can also initiate pyroptosis in a RAGE-dependent manner [144]. Additionally, RAGE activation also occurs via a membrane bound form of HMGB1, a process shown to be crucial for axonal sprouting and neurite outgrowth in vitro [145]. HMGB1 also binds the membrane PRR TLR4 and can initiate TNFα release from macrophages [146].

HMGB1 does not contain a leader sequence and thus is secreted via unconventional mechanisms independent of the ER/Golgi [147]. Secretion of HMGB1 occurs via both active and passive mechanisms. The first documented secretion of HMGB1 was observed by an active mechanism with relatively delayed kinetics compared to early pro-inflammatory cytokines IL-1β and TNFα [134]. In this pathway HMGB1 release occurs following generation of the bioactive lipid lysophosphatidylcholine (LPC) at the inflammatory site and is mediated by secretory lysosomes [148]. In this regard HMGB1 appears to behave more as a cytokine than a DAMP.

The active secretion of HMGB1 requires exclusion from the nucleus. This occurs due to acetylation of lysine residues, a process which prevents nuclear localisation and thus shifts the equilibrium of HMGB1 intracellular location towards the cytosol [129] (Figure 1). It was also observed that, upon acetylation by acetyltransferases such as P300/CBP-associated factor (PCAF), P300 or CREB-binding protein and nuclear exclusion, HMGB1 accumulates in secretory lysosomes ready for unconventional release from macrophages. Hyperacetylation of lysine residues can also modulate the ability of HMGB1 to bind and/or bend DNA [149]. Hyperactelyation of HMGB1 is not just modulated by increasing activity of acetyltransferases but also by decreasing activity of deacetylase enzymes histone deacetylase (HDAC)1, 4 and 5 [150,151] and of sirtuin-1 [152]. Although the translocation of HMGB1 into the cytosol requires acetylation there are also other factors involved. Poly(ADP)-ribose polymerase (PARP) is required for HMGB1 translocation from the nucleus in response to DNA-alkylating damage but only if HMGB1 is acetylated [153]. Further research has shown that, following stimulation of macrophages with LPS, extracellular signal-regulated kinase (ERK) signalling induced by reactive oxygen species leads to PARP-1 activation which then shifts the acetylation equilibrium of HMGB towards a more acetylated protein leading to translocation from the nucleus and into the cytoplasm (Figure 1) [154]. In addition to acetylation, phosphorylation has also been shown to play a key role in nuclear shuttling of HMGB1 [155] suggesting multiple modes of posttranslational modification may be important for its unconventional secretion. The above described mechanisms indicate how HMGB1 is relieved from nuclear encapsulation and can be actively secreted from live macrophage cells.

HMGB1 is also passively released from necrotic cells. This occurs due to the fact that HMGB1 only binds loosely to chromatin and thus can diffuse into the extracellular space upon loss of membrane integrity [156]. Necrotic release of HMGB1 occurs far more rapidly than the active secretion pathway. HMGB1 is also released during apoptotic cell death [135]. It was previously thought that HMGB1 was not released by apoptotic cells [156]. However, it has since been confirmed that HMGB1 is released in an “immunologically silent” form following inactivation by oxidation, a mechanism dependent on mitochondrial ROS [157].

HMGB1 is expressed constitutively in multiple cell types and is localised to the nucleus where it plays a major role in promoting gene transcription. In addition to this property, HMGB1 is unconventionally secreted either actively from live cells or passively as a result of necrosis. As a result of this, HMGB1 can induce an inflammatory response independently of its nuclear role and thus acts as a DAMP.

## 6. Conclusions

The host response to damage or infection relies on secretion/release of a complex combination of inflammatory factors through unconventional pathways. Some factors, such as the prototypical pro-inflammatory cytokine IL-1β, are not expressed within cells until stimulated by an inflammatory stimulus. Conversely, inflammatory mediators such as HMGB1 and IL-33 are constitutively expressed in multiple cell types and are ready for release. IL-1α appears to play both roles as it is suggested to be constitutively expressed in barrier cells such as the epithelium or endothelium but, similar to IL-1β, expression must be induced in myeloid cells.

IL-1α also appears to cross multiple classifications in terms of processing. Whilst the 31 kDa form is active at the IL-1 receptor, calpain-dependent cleavage to a 17 kDa form appears to substantially increase its potency. IL-1β on the other hand is only capable of activating IL-1R following inflammasome and caspase-1 dependent processing to a 17 kDa form. IL-33 is processed to an inactive form by caspase-1 whilst HMBG1 is not modified by processing but activity is heavily regulated by oxidation state.

IL-1β is also unique to the other proteins discussed here in subcellular distribution. IL-1β is distributed evenly across the cytosol whilst IL-1α, IL-33 and HMGB1 are all actively transported to the nucleus. These nuclear factors therefore hold dual functions with both nuclear and secretory roles. An additional dual role that is held by the factors discussed in this review is the property of both active and passive secretion. All four proteins discussed can be passively released upon cell death but also (to varying degrees and in varying cell types/stimuli) actively secreted by live cells. This suggests that there is an additional layer of dual functionality in unconventionally secreted proteins.

The reason for the differing properties amongst unconventionally secreted inflammatory proteins is not fully understood and the list of these proteins is far longer than the four discussed in in this review [158,159,160,161,162]. Amongst these other unconventionally secreted inflammatory proteins are the galectins (discussed previously), in particular galectin-3 (gal-3). Gal-3, unlike gal-1 [15], is secreted in a mechanism dependent on microvesicle shedding [163] and is highly expressed in monocytes, macrophages and dendritic cells [164,165]. Gal-3 is a potent proinflammatory immune modulator strongly associated with leukocyte recruitment [166,167], IL-1 production [168] and chemotaxis [169]. An additional inflammatory protein secreted via an unconventional pathway is interleukin (IL)-18. IL-18, initially described as “IFNγ-inducing factor” shares many properties with IL-1β and is secreted concomitantly with IL-1β following inflammasome activation [170] as well as after stimulation with cytokines such as IL-12 [162]. Like IL-1β, IL-18 is produced as a biologically inactive precursor form and is cleaved primarily by caspase-1 [171] but processing can also be dependent on other enzymes such as neutrophil proteinase-3 [172] or triggered by Fas ligand [173] independent of caspase-1. There also exists for IL-18 an endogenous regulatory mechanism called IL-18 binding protein [174] which is constitutively expressed and acts to dampen IL-18 signalling. The secretion of IL-18 is poorly understood, and it has been suggested that pro-IL-18 is released from dying cells and is processed extracellularly [175]. However, more evidence is required to confirm that cell death is absolutely necessary for IL-18 secretion and to uncover the exact mechanism by which IL-18 is released from the cell.

The reason for unconventional protein secretion is not fully understood. It is clear that unconventional secretion is absolutely necessary for function in the case of FGF2 as, when forced through the ER/Golgi pathway by addition of an FGF4 leader sequence, FGF2 failed to bind heparan sulphate due to deleterious posttranslational modifications [176]. This suggests that the unconventional secretory pathway allows cells to secrete proteins without the requirement for posttranslational modification. There have been similar suggestions relating to the secretion of IL-1β as, when forced through the ER/Golgi, IL-1β is *N*-glycosylated reportedly leading to loss of function [177,178].

It is perhaps more likely, however, that the reason for the unconventional secretion of inflammatory proteins, such as IL-1β, is in order to protect the host when conventional mechanisms are compromised. It has been observed that, under ER stress, cells remain capable of inflammasome activation (and IL-1 secretion) [179], and also that viral infections can dismantle the trans-Golgi network as a way of preventing host responses [180]. Cells with compromised secretion mechanisms may have severely impaired capability to counter infections if it were not for the ability to secrete proinflammatory factors, and thus mount an inflammatory response, through mechanisms independent of the ER/Golgi network.

Unconventional secretion also allows for proteins to hold roles within the cell additional to that of a classic proinflammatory cytokine. One such additional role, a particular focus of this review, is nuclear localisation. The reason for such contrasting roles to be held by the same protein remains largely unknown. One theory is that packaging in the nucleus prevents aberrant release of potentially damaging proinflammatory factors thus acting as a regulatory mechanism much in a similar fashion to IL-1β or IL-18 which require processing for activity. This is perhaps best exemplified by IL-33 which is highly damaging if excluded from the nucleus [119]. However, with factors such as HMGB1 this seems less likely as an active role within the nucleus is well defined. We hypothesise that the inflammatory function of HMGB1 is a more recent evolutionary modification to allow detection of uncontrolled cell death (in which the cell nucleus is ruptured and DNA is released) [181]. In this way, HMGB1 acts as a classical DAMP to alert the host to infection or damage that has compromised the nuclear membrane. In contrast to this, HMGB1 is also secreted through an active mechanism by live cells, implying it also possesses a signalling role independent of accidental cell death.

It would seem that in the case of IL-1α both of these properties are fulfilled as research has suggested that IL-1α is stored in the nucleus as a regulatory mechanism and that IL-1α acts within the nucleus to promote inflammation. Far more extensive research is required to fully elucidate the importance of the nuclear localisation of inflammatory cytokines.

All of the proteins discussed in this review are closely linked to non-communicable disease and thus may offer attractive targets for therapeutic inhibition. However, these factors also hold many other roles so care must be taken to not impair a vital cellular process or host response to infection when targeting. This emphasises the importance of further research to improve the understanding of how IL-1β, IL-1α, IL-33 and HMGB1 signal and are secreted as, in possession of this knowledge, we may be able to modulate specific roles of these factors (for example secretory cytokine role) without affecting homeostatic function (such as the nuclear role). This knowledge will be vital for the design of new therapeutics targeting inflammation.
